# Psychological impact of the COVID‐19 pandemic on infertile patients: A systematic review and meta‐analysis

**DOI:** 10.1002/pchj.782

**Published:** 2024-06-18

**Authors:** Yu Liu, Yiyao Su, Xiaoshan Li

**Affiliations:** ^1^ School of Psychology Jiangxi Normal University Nanchang China; ^2^ Center of Mental Health Education and Research Jiangxi Normal University Nanchang China

**Keywords:** COVID‐19 pandemic, infertile patient, meta‐analysis, psychological impact, review

## Abstract

The present study aimed to examine the psychological impact of the COVID‐19 pandemic on infertile patients. We adopted a comparison design and searched articles published from 1 September 2016 to 31 December 2019 as the control group, while articles published from 1 January 2020 to 31 April 2023 were treated as the pandemic group. Specifically, Web of Science, PubMed, Medline, PsycArticle, CNKI and PsycINFO were searched to identify potential eligible records. Risk of bias was assessed, and random‐effects meta‐analyses were conducted to estimate the prevalence of specific mental health problems. Forty studies with a total of 19,480 participants were included in the analysis. The pooled prevalence of anxiety in the pandemic group was significantly higher than that in the control group. The depression and stress prevalence in the pandemic group was higher than that in the control group, yet did not reach statistical significance. A subgroup analysis revealed region differences with developed countries exhibiting higher rates of anxiety and depression in the pandemic group, but the result was the opposite in the control group. Physiological factors, psychological factors and social factors correlated with infertile patients' mental health were identified. The COVID‐19 pandemic had a significant negative impact on infertile patients' mental health, emphasizing the importance of ways to mitigate the risks during the pandemic.

## INTRODUCTION

Infertile patients are vulnerable to mental health problems (Greil, 1997; Lawson et al., 2014; Lechner et al., 2007; Verhaak, Smeenk, Nahuis, et al., 2007). A number of studies indicate that a considerable proportion of infertile patients suffered from mental health disorders before (Holley et al., 2015; Pasch et al., 2016; Williams et al., 2007) and after COVID‐19 (Ben‐Kimhy et al., 2020; Boivin et al., 2020), such as anxiety and depression. Phenomenologically, the mental health problems among the infertile population seem to be worsened after the outbreak of COVID‐19. However, to our knowledge, the existing literature has not analyzed the difference in patients' mental health between the two periods, thus to reveal the psychological impact of the COVID‐19 pandemic on infertile patients. Therefore, the present study aimed to investigate the impact of COVID‐19 on infertile patients' mental health, and how the pandemic impacts on their mental health. Since anxiety, depression and stress are the most commonly used measurements of one's mental health (Peitz et al., 2021), we focused on the prevalence of anxiety, depression and stress among infertile patients in the present study.

Previous empirical research on infertile patients indicated mental health problems such as anxiety and depression, yet provided discrepant results. Take the pandemic period for example: some studies reported more than 70% of depression symptoms in infertile participants (Lawson et al., 2021; Schwab et al., 2022), while some other research showed less than 20% of depression prevalence (Barra et al., 2022; Kaur et al., 2020), and one study reported no clinical incident of depression (Lablanche et al., 2022). Considering the advantages of meta‐analysis over single investigation, such as practical significance and rigorous methodology (Shelby & Vaske, 2008), the present study adopted a meta‐analysis to draw a decisive conclusion on the magnitude of the impact of COVID‐19 on the psychological well‐being of infertile patients.

Given the possible negative impact of COVID‐19 on infertile patients, its underlying mechanism and difference in mental health‐related factors between pandemic and pre‐pandemic years remain unclear. To better explain the difference in infertile patients' mental health status, scholars explored factors associated with psychological outcomes from different perspectives under the physiological‐psychological‐social framework. Physiological factors include age (Barra et al., 2022; Gupta et al., 2021), gender (Barra et al., 2022; Esposito et al., 2020), duration of infertility (Esposito et al., 2020; Gordon & Balsom, 2020) and so on. For example, advancing age was found to be connected with higher anxiety levels (Barra et al., 2022). Psychological variables consist of personality (Gordon & Balsom, 2020), coping style (Seifer et al., 2021), previous psychiatric diagnosis (Lablanche et al., 2022) and so on. For example, one study found that coping strategies could mediate the relationship between intolerance of uncertainty and infertile women's level of distress (Mitrovic et al., 2022). Social factors include delay of fertility treatment (Lawson et al., 2021), decreased income (Cao et al., 2021), social support (Jaiswal et al., 2022) and so on. One study found that lower perceived social support was associated with higher psychological distress (Marom Haham et al., 2021). However, it still could not fully explain the difference in patients' mental health status between pandemic and pre‐pandemic years, as COVID‐19‐related factors may also play a role. Thus, COVID‐19 related unique stressors also need to be examined to help understand potential mechanisms. The identification of these variables could boost timely interventions and tailored mental health support, which is beneficial to higher resilience (Bao et al., 2020). Therefore, the second aim of this study was to explore potential variables associated with psychological outcomes in pandemic and pre‐pandemic years, thus to help understand how COVID‐19 affects infertile patients' mental health.

In conclusion, the present study aimed to understand the impact of the pandemic on the infertile population through comparing the prevalence of anxiety, depression and stress among infertile patients during and before the pandemic. The present study also aimed to identify potential factors associated with patients' psychological outcomes in pandemic and pre‐pandemic years, thus to further understand how COVID‐19 exerts its impact on infertile patients.

## MATERIALS AND METHODS

### Search strategy and study selection

Following the approval of the Ethical Review Board of the School of Psychology, Jiangxi Normal University, a systematic search of the literature was conducted in accordance with the Preferred Reporting Items for Systematic Reviews and Meta‐Analysis (PRISMA) statement and the Meta‐Analysis of Observational Studies in Epidemiology (MOOSE) guidelines (Moher et al., 2009; Stroup et al., 2000). Given that the COVID‐19 pandemic has lasted for three years since early 2020, we adopted comparison design and searched articles published from 1 September 2016 to 31 December 2019 as the control group. Literature published from 1 January 2020 to 31 April 2023 were set as the pandemic group. Specifically, Web of Science, PubMed, Medline, PsycArticle, CNKI and PsycINFO were searched to identify records that reported on the anxiety, depression or stress prevalence in infertile patients. Manual searches were conducted of the reference lists from retrieved articles and other systematic reviews investigating psychological outcomes in infertile patients. No language restrictions were applied. The following search items were used: (“infertile” or “infertility” or “childless”) and (“mental health” or “anxiety” or “depression” or “psychological” or “insomnia” or “stress” or “emotional” or “psychological adjustment”).

**FIGURE 1:**
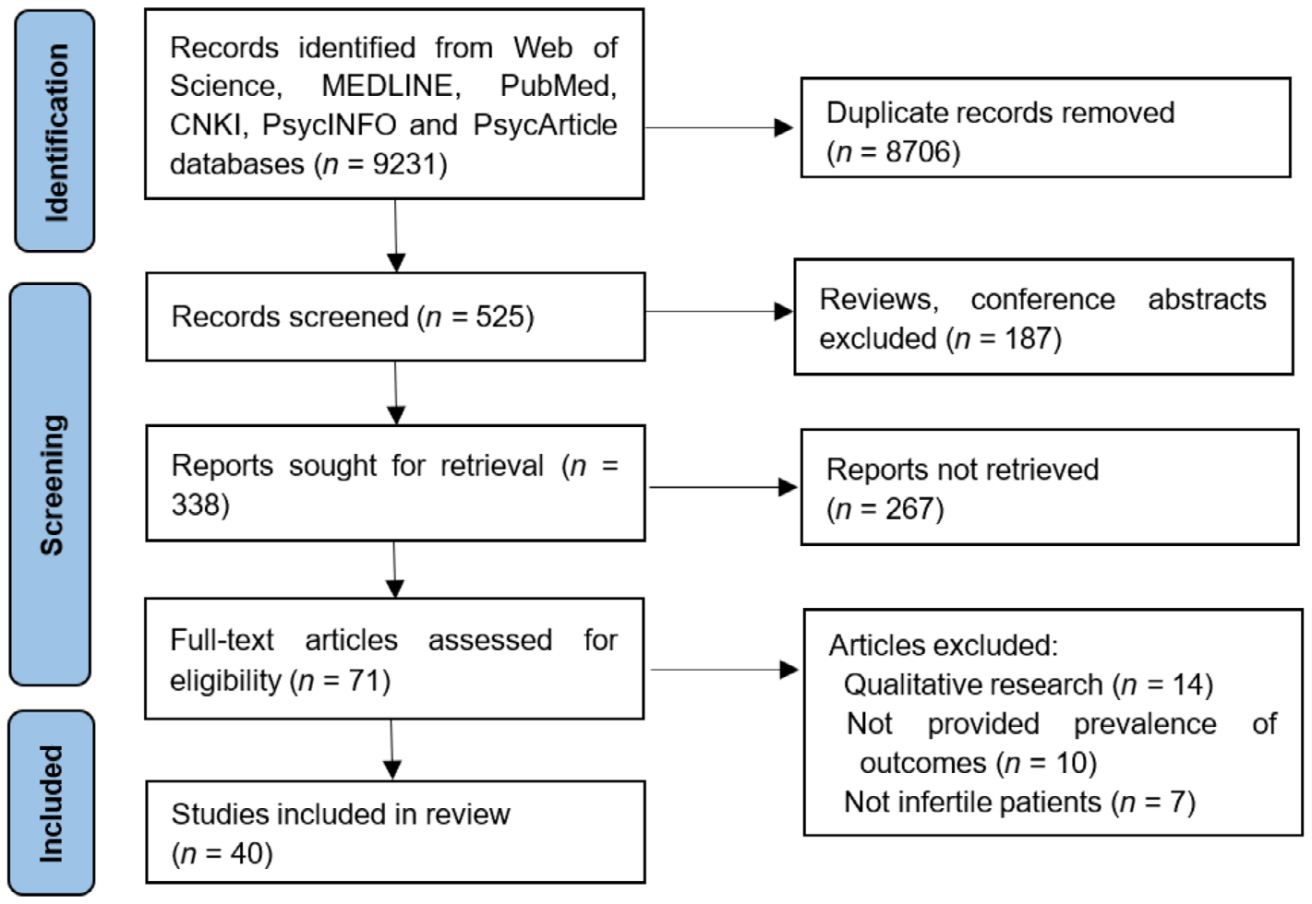
Flow chart of study selection process.

Articles were eligible if they met the following criteria: the study population group included infertile patients or infertile couples; the study reported prevalence of outcomes indicating the mental health of these patients; the study used validated assessment methods. We excluded studies with quantitative data (reviews, editorials, commentaries, qualitative researches). Conference presentations without information about the methods or outcomes were also excluded. Any disagreement in inclusion decisions was resolved by a third researcher. Figure 1 shows the complete study retrieval process.

### Data extraction

Two researchers independently extracted the following data from each article: study name; year of publication; sample size, including total number of participants and percentage of female participants; region; assessment methods; prevalence of anxiety, depression and stress; and factors associated with patients' psychological outcomes. Any disagreement on the extracted data was resolved through discussion.

### Assessment of study quality

The methodological quality of the included studies was assessed by two researchers independently using a modified form of the Newcastle‐Ottawa scale (Pappa et al., 2020). Assessment criteria include representativeness of sample, comparability between respondents and non‐respondents, assessment of outcome, sample size, and adequate statistics. The quality score ranged between 0 and 5. We assigned scores of ≥3 points as low risk of bias, while scores of <3 points were regarded as high risk of bias (Pappa et al., 2020). Possible disagreements were resolved by reaching a consensus through discussion. The results of the quality assessment and risk of bias are presented in Table 1.

**TABLE 1:** The modified Newcastle‐Ottawa score results.

Studies		Modified Newcastle‐Ottawa quality assessment scale					Score
		1	2	3	4	5	
**Pandemic group**							
Barra et al. (2022)		*	‐	*	*	*	4
Cao et al. (2021)		*	*	‐	*	‐	3
Dong, Tao, et al. (2021)		*	‐	‐	*	*	3
Dong, Wu, et al. (2021)		*	*	*	*	‐	4
Dong et al. (2022)		*	*	‐	*	*	4
Esposito et al. (2020)		*	*	‐	*	*	4
Gupta et al. (2021)		*	‐	*	‐	*	3
Gordon and Balsom (2020)		*	‐	‐	*	*	3
Jaiswal et al. (2022)		*	‐	‐	‐	*	2
Kaur et al. (2020)		*	‐	*	‐	*	3
Lablanche et al. (2022)		*	‐	‐	*	*	3
Lawson et al. (2021)		*	‐	‐	*	*	3
Li, Jin, et al. (2020)		*	‐	*	*	*	4
Rosielle et al. (2021)		*	‐	‐	‐	*	2
Schwab et al. (2022)		*	‐	‐	*	*	3
Tan (2022)		*	‐	*	‐	*	3
Zhang et al. (2023)		*	*	*	*	*	5
**Control group**							
Alshahrani et al. (2019)		*	‐	*	*	*	4
Bai et al. (2019)		*	*	‐	*	*	4
Biringer et al. (2018)		*	‐	‐	*	*	3
Bondade et al. (2018)		*	‐	‐	*	*	3
Crawford et al. (2017)		*	‐	‐	*	*	3
Hegyi et al. (2019)		*	‐	*	*	*	4
Salih Joelsson et al. (2017)		‐	‐	‐	*	*	2
Kaminskyi and Kolomiichenko (2018)		‐	‐	*	*	*	3
Kong et al. (2019)		*	‐	*	*	*	4
Lakatos et al. (2017)		*	‐	*	*	*	4
Li et al. (2017)		*	‐	*	*	*	4
Ma et al. (2017)		*	‐	‐	*	*	3
Madero et al. (2017)		*	‐	‐	*	*	3
Mahadeen et al. (2018)		*	‐	‐	*	*	3
Oladeji and OlaOlorun (2018)		*	‐	‐	*	*	3
Peloquin et al. (2018)		*	‐	‐	*	*	3
Omani Samani et al. (2017)		*	‐	‐	*	*	3
Vo et al. (2019)		*	‐	*	*	*	4
Wiweko et al. (2017)		*	‐	‐	*	*	3
Xu et al. (2017)		*	*	‐	*	*	4
Yang et al. (2017)		*	*	*	*	*	5
Yassa et al. (2019)		*	‐	*	*	*	4
Zaidouni et al. (2018)		*	‐	‐	*	*	3

*Note*: 1. Representativeness of sample; 2. Sample size >600 infertile patients; 3. Response rate >80%; 4. Validated assessment tools employed in the study; 5. Adequate statistics and no need for further calculations.

### Data analysis

Sensitivity analysis was performed by subtracting each study to assess whether it has an impact on the pooled prevalence of the remaining studies. Due to the discrepancy of patient populations, regions, and assessment methods across studies, we could not assume one true effect size; thus, a random‐effects model was used to calculate the pooled prevalence. *I* ^2^ >50% was regarded as high heterogeneity (Higgins et al., 2003). MetaXL (www.epigear.com) and the software Comprehensive Meta‐Analysis (CMA) were used to complete the computation of effect sizes. The main outcomes of this meta‐analysis were prevalence and confidence intervals (CI). Double arcsine method was applied to transform the proportions, in order to put due weight of the studies with prevalence close to 0 or 1 in the meta‐analysis (Barendregt et al., 2013). The pooled prevalence of psychological outcomes in the pandemic and control groups were calculated and further compared through the subgroup function in CMA in order to manifest the psychological impact of the COVID‐19 pandemic on infertile patients. Subgroup analyses were further performed by sex, different geographical regions, and assessment methods according to data availability. Meanwhile, we adopted a qualitative approach to analyze potential variables associated with psychological outcomes among infertile patients.

## RESULTS

### Study characteristics

After screening and assessment of eligibility, 17 studies with a total of 11,438 participants were eventually included in the pandemic group of the meta‐analysis (Barra et al., 2022; Cao et al., 2021; Dong, Tao, et al., 2021; Dong, Wu, et al., 2021; Dong et al., 2022; Esposito et al., 2020; Gordon & Balsom, 2020; Gupta et al., 2021; Jaiswal et al., 2022; Kaur et al., 2020; Lablanche et al., 2022; Lawson et al., 2021; Li, Jin, et al., 2020; Rosielle et al., 2021; Schwab et al., 2022; Tan, 2022; Zhang et al., 2023). All of the studies reported on the prevalence of anxiety, depression, or stress of infertile patients during the COVID‐19 pandemic. Nine of the studies were undertaken in developing countries, six of which were in China, while three took place in India. The remaining studies were undertaken in developed countries, most of which were in Europe (6/8, 75%). Among the 17 studies, eight included only female participants, eight included both males and females, and one study included only men. Table 2 lists the studies included in the analysis. Seven studies used self‐administered questionnaires, whose validation was confirmed as the author reported, whereas the other studies all utilized validated scales.

**TABLE 2:** Characteristics of included studies.

Author (publication year)	Country	Sample size (*n*)	Females %	Anxiety %	Depression %	Stress %	Factors associated with psychological outcomes
**Pandemic group**							
Barra et al. (2022)	Italy	524	58.8%	GAD‐7, 21.8%	PHQ‐9, 17.8%	NA	Female age; sex; partner suffered from psychological disorder
Cao et al. (2021)	China	759	100%	STAI, 15.2%	Self‐administered, 20.8%	NA	Decreased income; restricted activities; COVID‐19 infection
Dong, Tao, et al. (2021)	China	400	50%	GAD‐7, 27.8%	PHQ‐9, 30.3%	NA	
Dong, Wu, et al. (2021)	China	1442	57.4%	GAD‐7, 17.3%	PHQ‐9, 26.3%	Self‐administered, 26.8%	Interrupted fertility treatment
Dong et al. (2022)	China	940	65%	GAD‐7, 26.5%	NA	PSS‐14, 27.1%	
Esposito et al. (2020)	Italy	627	93.8%	STAI, 71.0%	NA	NA	Women; relative affected by COVID‐19
Gupta et al. (2021)	India	170	94.4%	Self‐administered, 59.0%	NA	NA	Advancing age; delay to treatment
Gordon and Balsom (2020)	Canada	92	100%	NA	PHQ‐9, 52.0%	NA	Duration of infertility; defensive pessimism; helplessness; avoidance; optimism; support seeking
Jaiswal et al. (2022)	India	250	100%	Self‐administered, 72.0%	NA	NA	Duration of infertility; worry about infection; partner support
Kaur et al. (2020)	India	86	94%	Self‐administered, 14.1%	Self‐administered, 17.2%	NA	Cancelation of fertility treatment
Lablanche et al. (2022)	France	421	100%	HADS‐A, 21.6%	HADS‐D, 0.0%	PSS‐10, 50.8%	A history of anxiety or depression; suspension of ART care
Lawson et al. (2021)	America	556	100%	GAD‐7, 71.5%	PHQ‐8, 77.7%	NA	Delayed fertility treatment; support seeking
Li, Jin, et al. (2020)	China	72	100%	SCL‐90, 66.7%	SCL‐90, 29.2%	NA	
Rosielle et al. (2021)	Netherlands	318	100%	NA	NA	Self‐administered, 76.6%	Increasing female age
Schwab et al. (2022)	German	274	100%	GAD‐2, 71.9%	PHQ‐2, 68.2%	NA	Reduced social network
Tan (2022)	Singapore	409	100%	NA	NA	Self‐administered, 49.4%	
Zhang et al. (2023)	China	4098	0%	GAD‐7, 36.3%	PHQ‐9, 39.6%	IES‐R, 6.7%	Previous psychiatric disorder
**Control group**							
Alshahrani et al. (2019)	Saudi Arabia	406	51%	MINI, 21.2%	MINI, 21.7%	NA	Monthly income
Bai et al. (2019)	China	740	51%	NA	PHQ‐9, 57.0%	NA	Social concern
Biringer et al. (2018)	Norway	467	100%	HADS, 20.1%	HADS, 7.7%	NA	
Bondade et al. (2018)	India	100	100%	HADS, 21.0%	HADS, 25.0%	NA	Intimate partner violence
Crawford et al. (2017)	America	416	100%	N.A.	PROMIS, 41.0%	NA	
Hegyi et al. (2019)	Hungary	113	N.A.	STAI, 4.9%	BDI, 4.5%	NA	Duration of infertility
Salih Joelsson et al. (2017)	Sweden	468	100%	HADS, 57.6%	EPDS, 15.7%	NA	Previous psychiatric diagnosis
Kaminskyi and Kolomiichenko (2018)	Ukraine	115	100%	STAI, 47.0%	BDI, 21.7%	SRRS, 47.8%	
Kong et al. (2019)	China	260	100%	SAS, 14.2%	SDS, 30.8%	NA	
Lakatos et al. (2017)	Hungary	134	100%	STAI, 39.6%	BDI, 44.8%	NA	ART history; social concern
Li et al. (2017)	China	211	100%	NA	SDS, 50.7%	NA	Social support
Ma et al. (2017)	China	245	0%	GAD‐7, 42.9%	PHQ‐9, 20.4%	NA	
Madero et al. (2017)	Spain	548	63%	HADS, 14.5%	HADS, 3.5%	NA	Sex; region
Mahadeen et al. (2018)	Jordan	248	58.5%	NA	BDI, 66.1%	NA	Sex
Oladeji and OlaOlorun (2018)	Nigeria	110	100%	NA	PHQ‐9, 52.7%	NA	Higher education level
Peloquin et al. (2018)	Canada	558	50%	PSI, 15.9%	PSI, 21.1%	NA	Self‐blame
Omani Samani et al. (2017)	Iran	360	50%	DASS, 29.2%	DASS, 35.8%	DASS, 28.9%	Hope
Vo et al. (2019)	Vietnam	401	100%	NA	PHQ‐9, 12.2%	NA	Previous antidepressant use
Wiweko et al. (2017)	Indonesia	63	100%	NA	NA	SRQ‐20, 22.3%	Duration of infertility
Xu et al. (2017)	China	842	100%	SAS, 21.3%	SDS, 21.3%	NA	Younger age; duration of infertility
Yang et al. (2017)	China	771	0%	STAI, 23.2%	MHI‐5, 36.2%	NA	Duration of infertility
Yassa et al. (2019)	Turkey	226	100%	HADS, 21.7%	BDI, 15.5%	NA	Treatment attempt
Zaidouni et al. (2018)	Morocco	240	50%	NA	NA	PSS‐10, 47.1%	Duration of infertility; higher education level

Twenty‐three studies with a total of 8042 participants were included in the control group (Alshahrani et al., 2019; Bai et al., 2019; Biringer et al., 2018; Bondade et al., 2018; Crawford et al., 2017; Hegyi et al., 2019; Kaminskyi & Kolomiichenko, 2018; Kong et al., 2019; Lakatos et al., 2017; Li et al., 2017; Ma et al., 2017; Madero et al., 2017; Mahadeen et al., 2018; Oladeji & OlaOlorun, 2018; Omani Samani et al., 2017; Peloquin et al., 2018; Salih Joelsson et al., 2017; Vo et al., 2019; Wiweko et al., 2017; Xu et al., 2017; Yang et al., 2017; Yassa et al., 2019; Zaidouni et al., 2018). The details of the included studies are summarized in Table 2.

### Anxiety prevalence

**FIGURE 2:**
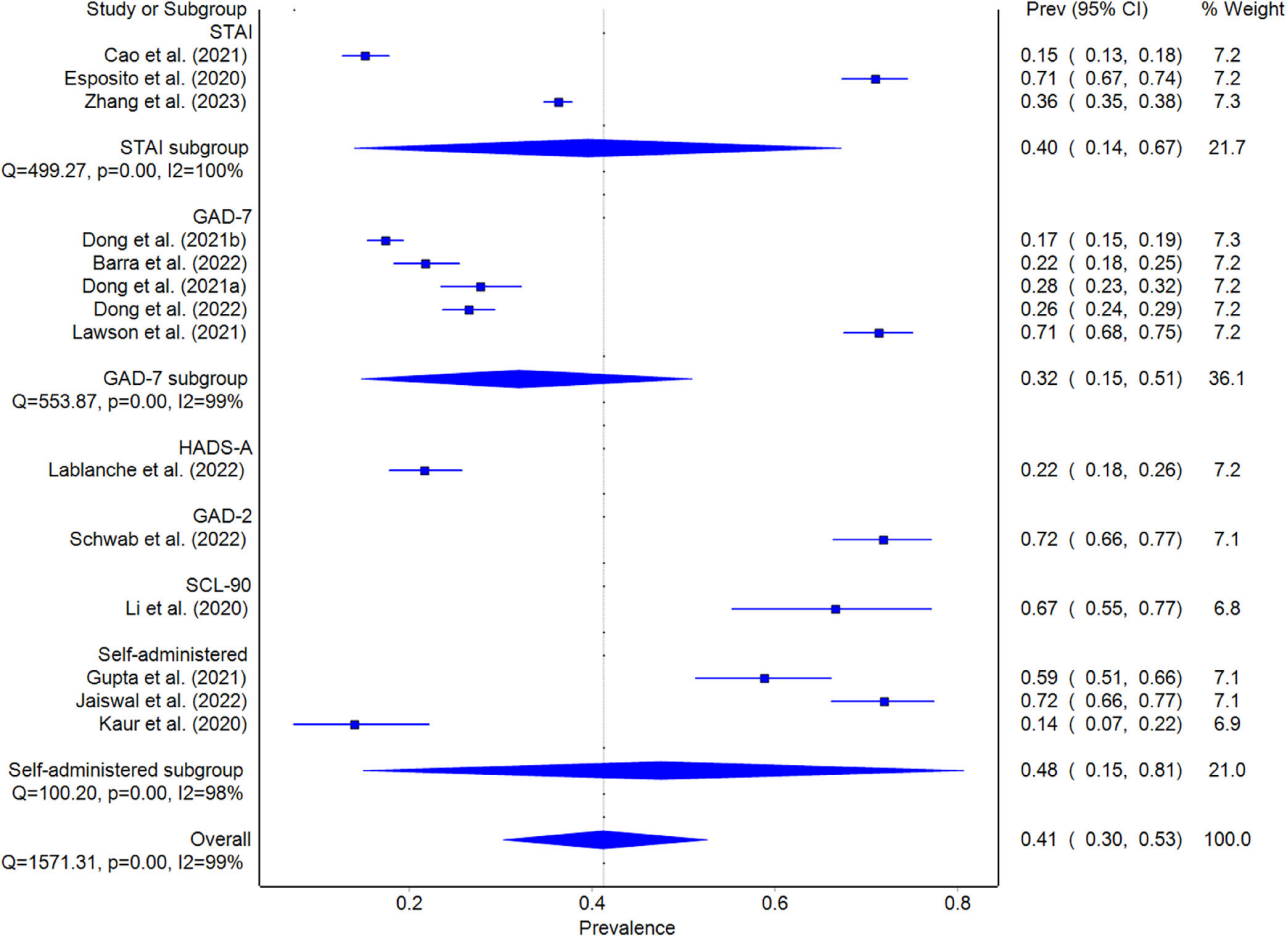
Pooled anxiety prevalence by assessment method in pandemic group.

In the pandemic group, anxiety prevalence was analyzed in 14 studies (Barra et al., 2022; Cao et al., 2021; Dong, Tao, et al., 2021; Dong, Wu, et al., 2021; Dong et al., 2022; Esposito et al., 2020; Gupta et al., 2021; Jaiswal et al., 2022; Kaur et al., 2020; Lablanche et al., 2022; Lawson et al., 2021; Li, Jin, et al., 2020; Schwab et al., 2022; Zhang et al., 2023). The pooled anxiety prevalence was 41.2% (95% CI = 30.3–52.6, *I* ^2^ = 99%) as shown in Figure 2. Sensitivity analysis indicated that no study affected the pooled anxiety prevalence by more than 3% when excluded.

**FIGURE 3:**
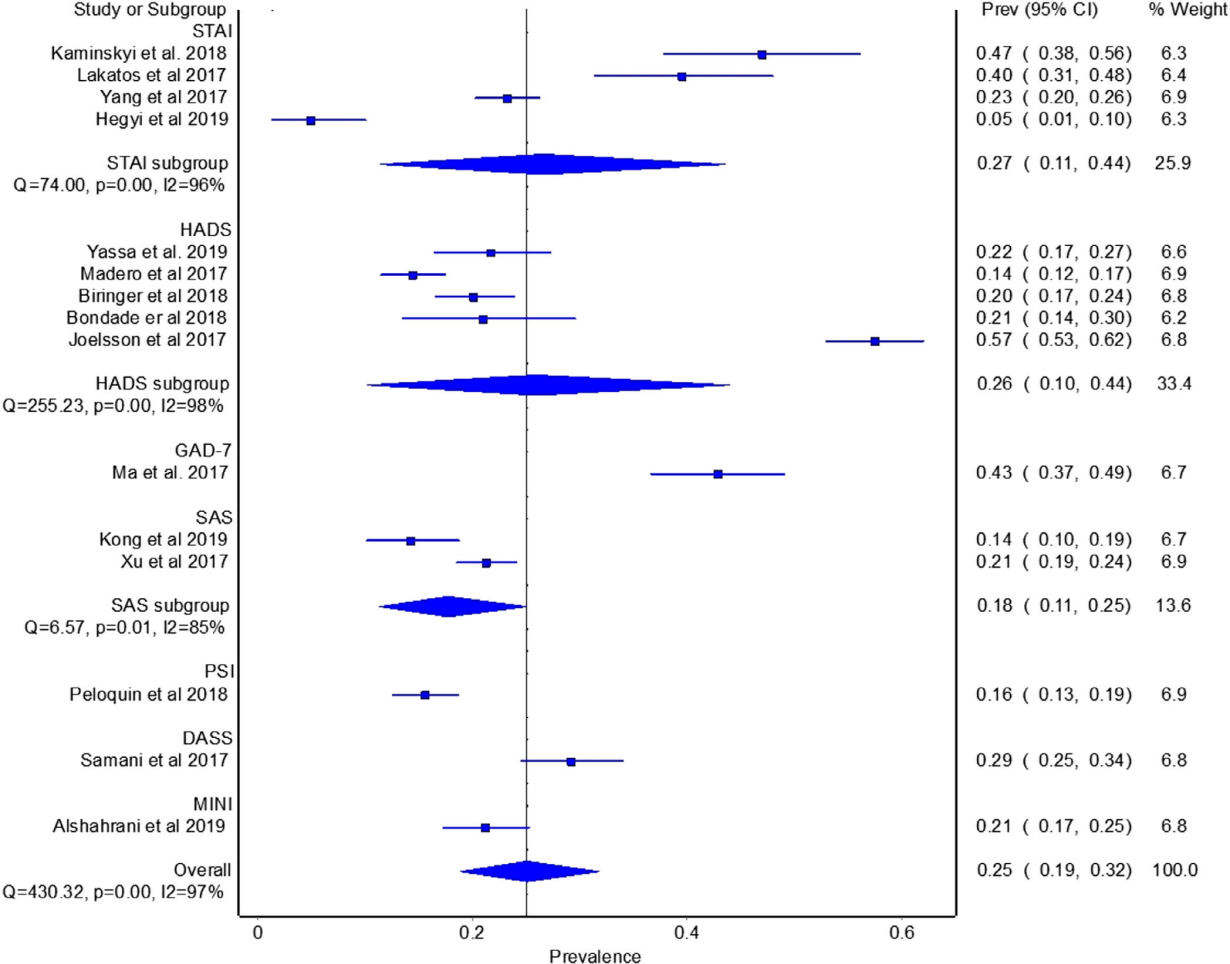
Pooled anxiety prevalence by assessment method in control group.

Fifteen studies (Alshahrani et al., 2019; Biringer et al., 2018; Bondade et al., 2018; Hegyi et al., 2019; Kaminskyi & Kolomiichenko, 2018; Kong et al., 2019; Lakatos et al., 2017; Ma et al., 2017; Madero et al., 2017; Omani Samani et al., 2017; Peloquin et al., 2018; Salih Joelsson et al., 2017; Xu et al., 2017; Yang et al., 2017; Yassa et al., 2019) reported data on anxiety in the control group, with a pooled prevalence of 25.2% (95% CI = 19.3–31.7, *I* ^2^ = 96%) (see Figure 3). No study affected the pooled anxiety prevalence by more than 3% when excluded. Anxiety prevalence in pandemic years among infertile patients was significantly higher than in pre‐pandemic years (41.2% vs. 25.2%, *Q* = 5.928, *p < *.05).

### Depression prevalence

**FIGURE 4:**
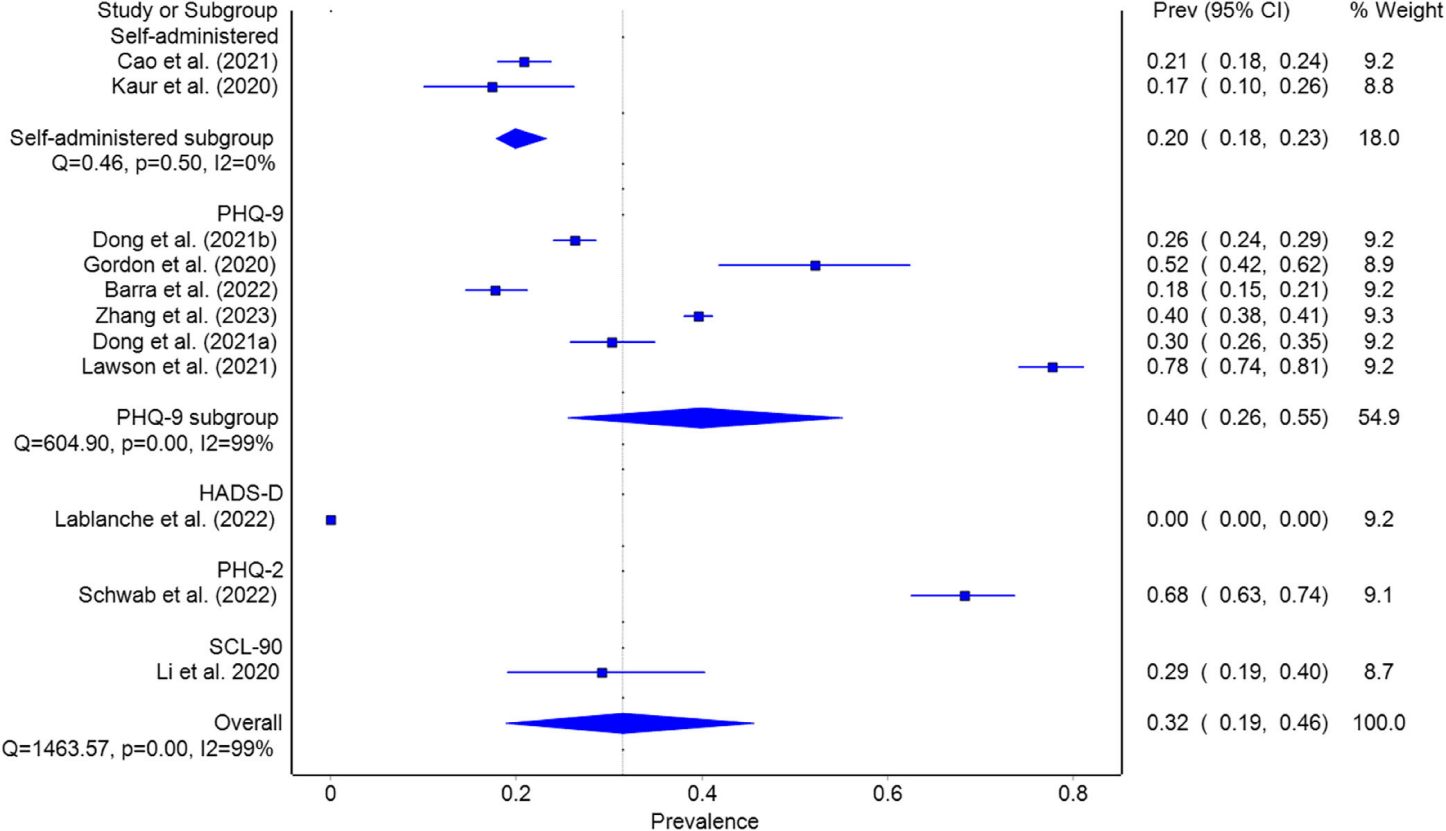
Pooled depression prevalence by assessment method in pandemic group.

Depression prevalence was estimated in 11 studies within the pandemic group (Barra et al., 2022; Cao et al., 2021; Dong, Tao, et al., 2021; Dong, Wu, et al., 2021; Gordon & Balsom, 2020; Kaur et al., 2020; Lablanche et al., 2022; Lawson et al., 2021; Li, Jin, et al., 2020; Schwab et al., 2022; Zhang et al., 2023), with a pooled prevalence of 31.5% (95% CI = 18.9–45.6, *I* ^2^ = 99%) as presented in Figure 4. The only study to affect the outcome by more than 3% was Lablanche et al. (2022); when excluded, the recalculated pooled prevalence was 37.3% (95% CI = 26.1–49.1, *I* ^2^ = 98%).

**FIGURE 5:**
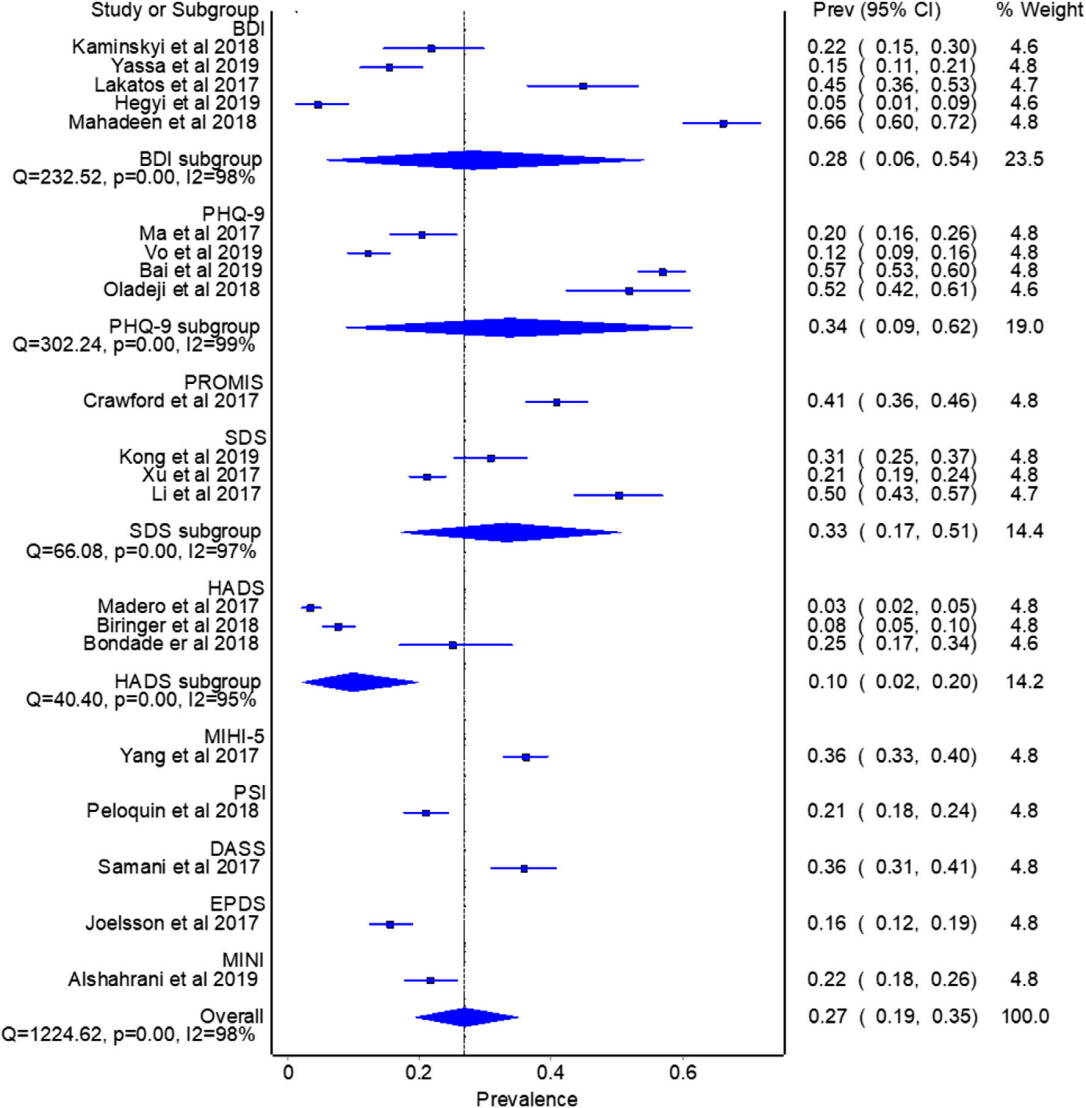
Pooled depression prevalence by assessment method in control group.

In the control group, depression data were available in 21 studies (Alshahrani et al., 2019; Bai et al., 2019; Biringer et al., 2018; Bondade et al., 2018; Crawford et al., 2017; Hegyi et al., 2019; Kaminskyi & Kolomiichenko, 2018; Kong et al., 2019; Lakatos et al., 2017; Li et al., 2017; Ma et al., 2017; Madero et al., 2017; Mahadeen et al., 2018; Oladeji & OlaOlorun, 2018; Omani Samani et al., 2017; Peloquin et al., 2018; Salih Joelsson et al., 2017; Vo et al., 2019; Xu et al., 2017; Yang et al., 2017; Yassa et al., 2019) with a pooled prevalence of 26.9% (95% CI = 19.4–53.1, *I* ^2^ = 98%) as shown in Figure 5. No study affected the pooled depression prevalence by more than 3% when excluded. Depression prevalence in pandemic years among infertile patients was higher than in pre‐pandemic years. However, it did not reach statistical significance (37.3% vs. 26.9%, *Q* = 2.448, *p = *.118)

### Stress prevalence

**FIGURE 6:**
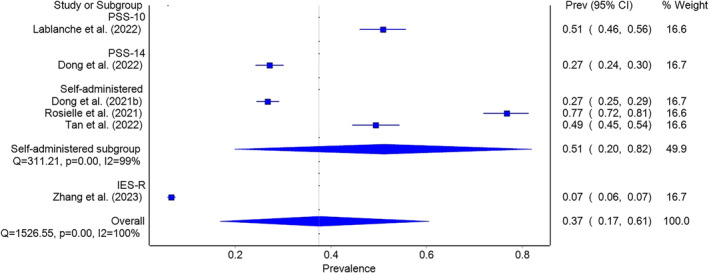
Pooled stress prevalence by assessment method in pandemic group.

Stress prevalence was assessed in six (Dong, Wu, et al., 2021; Dong et al., 2022; Lablanche et al., 2022; Rosielle et al., 2021; Tan, 2022; Zhang et al., 2023) out of the 17 included studies (see Figure 6). The pooled prevalence was 37.5% (95% CI = 16.8–60.5, *I* ^2^ = 99%). In sensitivity analysis, two studies (Rosielle et al., 2021; Zhang et al., 2023) affected the result by over 5%; when excluded, the recalculated pooled prevalence was 37.9% (95% CI = 26.0–50.5, *I* ^2^ = 97%).

**FIGURE 7:**
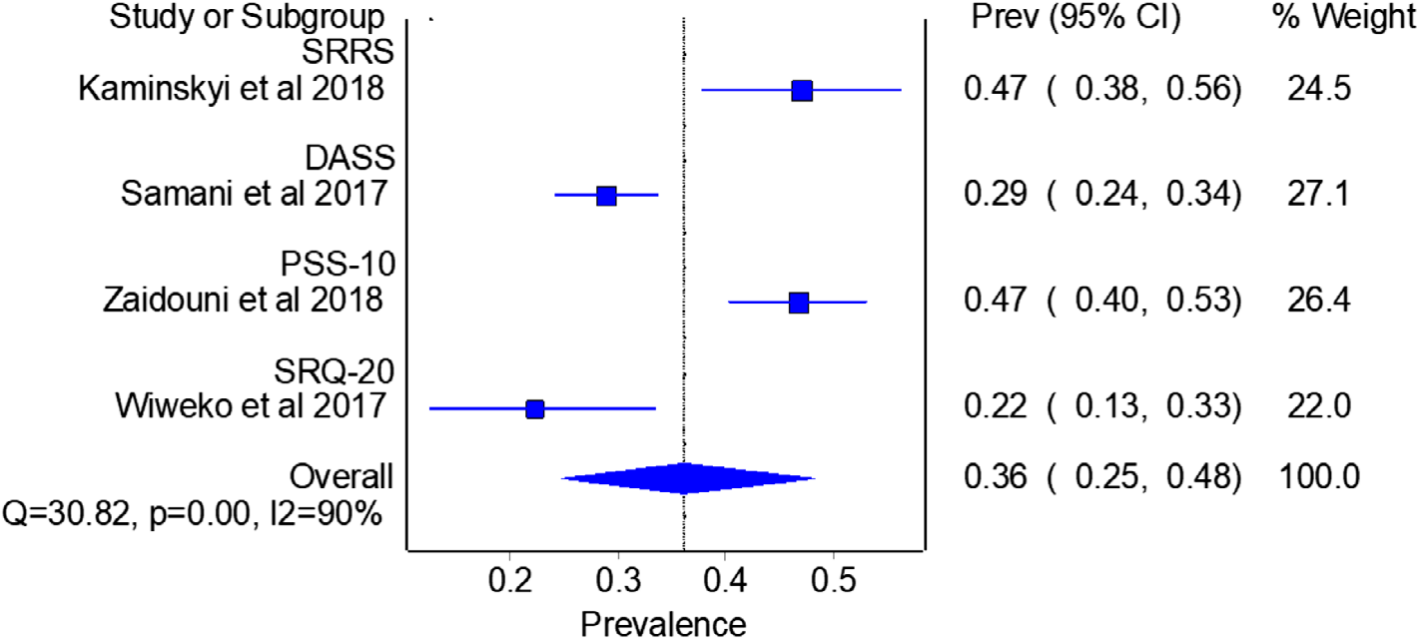
Pooled stress prevalence by assessment method in control group.

As for the control group, stress prevalence was analyzed in four studies (Kaminskyi & Kolomiichenko, 2018; Omani Samani et al., 2017; Wiweko et al., 2017; Zaidouni et al., 2018), with a pooled prevalence of 36.1% (95% CI = 24.8–48.2, *I* ^2^ = 90%) as shown in Figure 7. We found no significant difference in stress prevalence between pandemic and pre‐pandemic years in infertile patients (37.9% vs. 36.1%, *Q* = 0.016, *p = *.898). The comparison of pooled anxiety, depression and stress prevalence between pandemic and pre‐pandemic years is summarized in Table 3.

**TABLE 3:** Comparison of pooled anxiety, depression and stress prevalence between pandemic and pre‐pandemic years.

Group	Anxiety	Depression	Stress
Pandemic	41.20%	37.30%	37.90%
Pre‐pandemic	25.20%	26.90%	36.10%
*Q*	5.928	2.448	0.016
*p*‐value	*p* < .05	*p* = .118	*p* = .898

### Subgroup analysis by sex and region

Subgroup analysis of anxiety and depression prevalence by sex and region was conducted as presented in Table 4.

**TABLE 4:** Subgroup analysis of anxiety and depression prevalence.

Factor	Subgroup	Anxiety		Depression	
		Pandemic	Pre‐pandemic	Pandemic	Pre‐pandemic
Gender	Female	40.3%	27.3%	25.6%	27.2%
	95% CI	24.1‐57.6	19.3‐36.2	7.6‐49.1	18.6‐36.7
	*I* ^2^	98%	96%	99%	97%
	Male	36.8%	23.8%	27.4%	25.1%
	95% CI	28.1‐45.9	14.3‐34.8	13.1‐46.3	12.3‐40.4
	*I* ^2^	93%	95%	97%	97%
Region	Developing country	35.6%	26.0%	27.2%	32.3%
	95% CI	25.6‐46.3	20.8‐31.6	19.7‐35.4	23.7‐41.5
	*I* ^2^	98%	91%	97%	97%
	Developed country	51.3%	22.8%	30.6%	16.6%
	95% CI	25.5‐76.7	9.6‐39.2	0.0‐84.3	6.8‐29.3
	*I* ^2^	99%	98%	99%	98%

In pandemic years, the pooled anxiety prevalence was 40.3% for females and 36.8% for males. In developing countries, the pooled anxiety prevalence was 35.6%, compared with 51.3% in developed countries. In pre‐pandemic years, the respective values of pooled anxiety prevalence for females and males were 27.3% and 23.8%. The pooled anxiety prevalence in developing and developed countries were 26.0% and 22.8%, respectively.

As for depression, sex data were also calculated in both groups, with a pooled prevalence of 25.6% for females and 27.4% for males in pandemic years. Accordingly, the pooled depression prevalence was 27.2% for females and 25.1% for males in pre‐pandemic years. Between different living areas, the pooled depression prevalence in developing countries was 27.2%, compared with 30.6% in developed countries in pandemic years. In contrast, the pooled prevalence of depression in developing countries and developed countries were 32.3% and 16.6%, respectively, in pre‐pandemic years.

For stress prevalence, subgroup analysis was not conducted because of the limited data reported.

### Factors associated with psychological distress among infertile patients

We adopted the physiological‐psychological‐social perspective to analyze the factors associated with infertile patients' psychological outcomes in both groups (see Table 5). Meanwhile, we recognized risk and protective factors under this framework.

**TABLE 5:** Factors associated with psychological outcomes in both groups.

Factor	Pandemic	Pre‐pandemic
Physiological factors	Advancing age (+)	Younger age (+)
	Duration of infertility (+)	Duration of infertility (+)
	Infertility diagnosis (+)	A history of ART treatment (+)
	Female sex (+)	Female sex (+)
Psychological factors	Optimism (−)	Previous psychiatric diagnosis (+)
	Defensive pessimism (+)	Hope (−)
	Helplessness (+)	
	Worry about getting infection (+)	
	Avoidance coping (+)	
	Previous psychiatric diagnosis (+)	
	Support seeking (−)	
	High resilience (−)	
Social factors	Delay of fertility treatment (+)	Social concern (+)
	Decreased income (+)	Higher education level (+)
	Restricted activities or communications (+)	Social support (−)
	Increasing COVID‐19 infections (+)	
	Relative affected by COVID‐19 (+)	
	Reduced social network due to lockdown (+)	
	Partner suffered from psychological disorder (+)	
	Spousal support (−)	

*Note*: +, positively correlated with psychological distress; −, negatively correlated with psychological distress.

As for physiological factors, the pandemic group and the control group both analyzed variables of age, sex and duration of infertility. In the pandemic group, advancing age, especially infertile women over 35 years old, was found to be a risk factor on infertile patients' mental health (Barra et al., 2022; Gupta et al., 2021; Rosielle et al., 2021), while younger age was associated with higher prevalence of mental disorders in the control group (Xu et al., 2017). Duration of infertility and female sex were two common risk factors in both groups (Barra et al., 2022; Esposito et al., 2020; Gordon & Balsom, 2020; Hegyi et al., 2019; Jaiswal et al., 2022; Madero et al., 2017; Mahadeen et al., 2018; Wiweko et al., 2017; Yang et al., 2017; Zaidouni et al., 2018). In addition, a history of antiretroviral therapy (ART) treatment (Lakatos et al., 2017; Yassa et al., 2019) was recognized as a risk factor in the control group.

As regards psychological factors, some personality characteristics (e.g., defensive pessimism) were predictors of poorer mental health, while optimism (Gordon & Balsom, 2020) and high resilience (Schwab et al., 2022) was predictive of more favorable outcomes. Negative emotions (e.g., sense of helplessness) and avoidance coping were recognized as risk factors (Gordon & Balsom, 2020; Jaiswal et al., 2022). In contrast, hope (Omani Samani et al., 2017) and support seeking (Gordon & Balsom, 2020; Lawson et al., 2021) were two protective factors. Moreover, infertile patients with a previous psychiatric diagnosis tended to have higher prevalence of mental health problems in both groups (Lablanche et al., 2022; Salih Joelsson et al., 2017; Vo et al., 2019; Yang et al., 2017; Zhang et al., 2023).

The pandemic group and the control group reported heterogeneous social factors. The most common risk factor reported in the pandemic group was delay or suspension of fertility treatment (Dong, Wu, et al., 2021; Gordon & Balsom, 2020; Gupta et al., 2021; Jaiswal et al., 2022; Kaur et al., 2020; Lablanche et al., 2022; Lawson et al., 2021). In addition, decreased economic income, restricted activities and communications, and the growing number of COVID‐19 infections were found to be three major sources of stress among quarantined patients (Cao et al., 2021). Other risk factors include relative affected by COVID‐19 (Esposito et al., 2020), reduced social network (Schwab et al., 2022), partner suffered from psychological disorder (Barra et al., 2022). Meanwhile, spousal support was correlated with lower perceived stress levels (Jaiswal et al., 2022). In the control group, social concern (Bai et al., 2019; Lakatos et al., 2017) and higher education level (Oladeji & OlaOlorun, 2018; Zaidouni et al., 2018) were correlated with higher distress level, while social support also had a protective effect against mental health problems (Li et al., 2017).

## DISCUSSION

This systematic review and meta‐analysis examined existing evidence on the psychological impact of the COVID‐19 pandemic on the infertile population. Specifically, pooled prevalence of anxiety, depression and stress were calculated and compared between pandemic years and pre‐pandemic years. Factors associated with psychological outcomes among infertile patients were analyzed under physiological‐psychological‐social framework. Study findings could contribute to deeper understanding of the psychological impact of COVID‐19 on infertile patients and boost tailored psychological interventions, which is beneficial to strengthen patients' mental health under the COVID‐19 pandemic.

### Prevalence difference between pandemic years and pre‐pandemic years

The present study meta‐analyzed the prevalence of anxiety, depression and stress under pandemic and pre‐pandemic years through a comparison design. Study findings indicate that infertile patients' anxiety prevalence in pandemic years was significantly higher than in pre‐pandemic years. Depression and stress prevalence in pandemic years were higher than in pre‐pandemic years, yet statistical significance was not reached.

Previous research showed that the prevalence rates of anxiety and depression of health care workers during the pandemic were 23.2% and 22.8% respectively, while the rates in the general population ranged between 6.33% and 50.9% for anxiety, 14.6% and 48.3% for depression, and 8.1% and 81.9% for stress (Pappa et al., 2020; Xiong et al., 2020). Compared to previously reported outcomes, the prevalence of anxiety, depression and stress of infertile patients (41.6%, 36.8% and 37.9%, respectively) are at the higher end, which shows the significant effect of the crisis on the mental health of the infertile population.

The difference between anxiety prevalence of the two groups could be first explained by the serious threat to one's health, especially the potential influence of the virus on the human reproduction system, caused by the new virus because of its high infectivity and mortality (Chandi & Jain, 2021; Huang et al., 2020; Li, Chen, et al., 2020; Patel et al., 2021). Previous research also showed that the onset of global pandemic diseases can cause an atmosphere of anxiety (Wang et al., 2020). Secondly, the cancellation or suspension of fertility care during the COVID‐19 pandemic may also be responsible. The closure of fertility clinics was appraised as a threat to the goal of being a parent (Boivin et al., 2020), especially for infertile women of advancing age. According to Ben‐Kimhy et al. (2020), fertility patients faced huge emotional burden (e.g., helplessness and sadness) because of the cancellation of cycles of fertility treatment in addition to the general distress during the pandemic. Thirdly, researchers have found that social support can lead to less anxiety and more resilience (Landman‐Peeters et al., 2005; Seifer et al., 2021). However, infertile patients' support systems may have been weakened due to social distancing or isolation, thus leading to more incidence of anxiety disorders. Therefore, infertile patients can have higher levels of anxiety during the COVID‐19 pandemic.

The stress prevalence in the pandemic years was slightly higher than in pre‐pandemic years, while no significant difference was found. According to previous studies, the success rate of assisted reproductive techniques remains low in certain groups of infertile patients, which could cause significant psychological burden (Miron‐Shatz et al., 2021). The literature has also found that fertility treatment is consistently related with infertility stress (Domar et al., 2012; Esteves et al., 2020; Pook & Krause, 2005; Verhaak, Smeenk, Evers, et al., 2007). Therefore, it is likely that fertility patients have similar stress levels whether in the period of pandemic breakout or not.

### Prevalence difference between different sexes and regions

Regarding sex difference, the subgroup analysis revealed that the anxiety prevalence appeared to be slightly higher in females in both groups. Meanwhile, females and males appeared to have similar prevalence of depression in both pandemic years and pre‐pandemic years. This finding reflects that both infertile men and women were significantly affected by the pandemic, which weakened the sex difference in the impact of infertility on patients. The subgroup analysis of our study showed a converse result regarding regional difference. In pre‐pandemic years, the anxiety and depression prevalence in developing countries were higher than in developed countries. This may be attributed to the better economic and medical conditions in developed countries, which leads to higher odds of successful pregnancy (Rainer et al., 2011). However, the result turned out opposite in pandemic years. Notably, six of the nine studies in developing countries were conducted in China, in which the public had a good sense of security due to the control of the virus spreading and the extensive COVID‐19 vaccination. Patients may also benefit from Confucianism, which argues that adversity or stress can help individuals' growth, thus leading to lower levels of anxiety and depression (Jing, 2006).

### Factors associated with prevalence of mental health disorders

The present study recognized factors associated with infertile patients' mental health in pandemic and pre‐pandemic years. Common associating factors were found according to the physiological‐psychological‐social framework. For physiological factors, duration of infertility and age were consistently correlated with higher prevalence of depression and anxiety regardless of the existence of the COVID‐19 pandemic. Common psychological factors include previous psychiatric diagnosis, which is shown to be negatively related with patients' mental health. Apart from anxiety, depression and stress, a large proportion of psychiatric patients also suffered from moderately severe to severe insomnia along with potential post‐traumatic stress disorder (PTSD) (Hao et al., 2020). In addition, optimism, high resilience and hope should also be considered common protective factors despite the search limitations applied in this article. Regarding social factors, social support was found to be a common protective factor against mental health problems in both groups. However, we found a difference in how these factors influence patients' mental health. For example, we found that advancing age was related with higher levels of distress during the pandemic, while younger age was associated with poor mental health in pre‐pandemic years. During the pandemic, fertility treatment was largely delayed or suspended due to the global outbreak of COVID‐19, which may lead to decreased odds of successful pregnancy among infertile women of advancing age (Schmidt et al., 2012). In contrast, younger patients may have more mental health problems in pre‐pandemic years because of insufficient experience and lack of cognitive preparations.

Unique stressors related to COVID‐19 were also identified through systematic review. Notably, financial concerns appeared to have an increased impact on patients' mental health during the pandemic, reminding of the importance of financial support when fertility treatment resumed (Ikeda et al., 2022). As the pandemic broke out, the physical condition of patients and their family members or relatives can also be predictive of their psychological status. The study found that concern about other family members getting COVID‐19 was significantly associated with stress and anxiety levels (Cao et al., 2021), suggesting that the COVID‐19 pandemic affected patients' mental health not only through themselves, but through their social network. Research also showed that reduced social network during the pandemic was positively associated with psychological distress (Schwab et al., 2022). When the number of COVID‐19 infections increase sharply, people would face quarantine or restriction of activities, which may lead to higher levels of distress (Brooks et al., 2020; Chen et al., 2022; Pfefferbaum & North, 2020). Therefore, the COVID‐19 related stressors may be the important cause for the poor mental health condition among infertile patients. In this case, online or internet‐based psychological intervention is vital to protect infertile patients from worsened mental health conditions. Previous studies have found internet cognitive behavioral therapy (I‐CBT) to be safe and efficacious for mental health problems, such as anxiety, insomnia and depression (Carl et al., 2020), which could be implemented through various online platforms. Mindfulness‐based cognitive therapy was also found to be helpful for stress‐related issues.

## STRENGTHS AND LIMITATIONS

To our knowledge, this is the first meta‐analysis and systematic review to adopt a comparison design to analyze the psychological impact of the COVID‐19 pandemic on infertile patients. Although the number of studies included in our meta‐analysis was relatively low, our results provide valuable insights into potential specific vulnerabilities among the infertile population. Findings on the prevalence difference of mental disorders between pandemic and pre‐pandemic years revealed the significant negative impact of the pandemic on infertile patients' mental health. The identification of unique stressors related to COVID‐19 can help better explain how COVID‐19 affects patients' psychological status, which is beneficial to enhance individual understanding of COVID‐19 and its consequences. In addition, it also provides positive value to the psychological interventions of infertile patients' mental health under epidemic background similar to COVID‐19.

Our meta‐analysis has some limitations. First, most of the included participants were infertile men or women who were seeking medical help at fertility centers. For those infertile individuals who did not seek medical help, we were not able to analyze their mental health status. Second, we applied no language restriction in this meta‐analysis, but most of the included studies were English and Chinese except one in Ukrainian due to our limited resources. Therefore, we may have overlooked studies written in other languages. Third, some research reported data in other forms (e.g., mean and standard error) rather than prevalence, and thus were not suitable for inclusion, which may have an influence on our results. Fourth, longitudinal studies may be a better way to answer our study question. Despite the above limitations, this meta‐analysis and systematic review should help us to have a more comprehensive understanding of the psychological impact of the COVID‐19 pandemic on infertile patients across regions, ethnicities and sex. Studies from various regions and studies focusing on those infertile patients who are not seeking medical help are required to better characterize their mental health in the future.

## CONCLUSIONS

The COVID‐19 pandemic had a significant negative impact on infertile patients' mental health status, causing a high prevalence of anxiety, depression and stress among this population. Infertile patients are more psychologically distressed under pandemic conditions, especially those living in developed countries. The findings of this study can contribute to tailored psychological interventions that enhance mental health among the infertile population.

## CONFLICT OF INTEREST STATEMENT

The authors declare no potential conflicts of interest.

## ETHICS STATEMENT

The research was conducted in compliance with APA’s ethical standards and the study was approved by the Ethical Review Board of the School of Psychology, Jiangxi Normal University.
